# Physical and 3D Printing Properties of Arrowroot Starch Gels

**DOI:** 10.3390/foods11142140

**Published:** 2022-07-19

**Authors:** Meiling Xu, Qiaoru Dong, Guiying Huang, Ya Zhang, Xuanxuan Lu, Jiaduo Zhang, Kun Zhang, Qingrong Huang

**Affiliations:** 1 School of Biotechnology and Health Sciences, Wuyi University, Jiangmen 529000, China; xumeiling95@163.com (M.X.); zhangya19960227@163.com (Y.Z.); zjiaduo@163.com (J.Z.); kzhang@gdut.edu.cn (K.Z.); 2 Department of Food Science, Rutgers University, 65 Dudley Road, New Brunswick, NJ 08901, USA; qd44@scarletmail.rutgers.edu; 3 College of Light Industry and Food Science, Zhongkai University of Agriculture and Engineering, Guangzhou 510225, China; 4 Department of Food Science and Engineering, Jinan University, Guangzhou 510632, China; luxuanxuan2@jnu.edu.cn

**Keywords:** arrowroot starch, microstructure, rheological properties, 3D printing

## Abstract

This paper aims to investigate the physical and 3D printing properties of arrowroot starch (AS), a natural biopolymer with many potential health benefits. Scanning electron microscopy images showed that AS granules had mixed spherical and elongated geometries, with average sizes of 10.5 ± 2.5 μm. The molecular weight of AS measured by gel permeation chromatography (GPC) was 3.24 × 10^7^ g/mol, and the amylose/amylopectin ratio of AS was approximately 4:11. AS has an A-type crystal structure, with a gelatinization temperature of 71.8 ± 0.2 °C. The overlap concentration (C*) of AS in aqueous solutions was 0.42% (*w/v*). Temperature-dependent dynamic rheological analyses of 10% to 30% (*w/v*) AS fluids showed that the storage modulus (G’) reached the maximum values around the gelatinization temperatures, while the yield stress (τ_y_) and flow stress (τ_f_) values all increased with the increase in AS concentration. The printing accuracy of AS gels was found to be associated with the interplay between the G’ values and the restorability after extrusion, determined by the three-interval thixotropy tests (3ITT). The optimum 3D printing condition occurred at 20% (*w/v*) AS, the nozzle diameter of 0.60 mm, the printing speed of 100 mm/s and the extrusion speed of 100 mm/s. Our research provides a promising biopolymer to be used in the design of novel personalized functional foods.

## 1. Introduction

*Maranta arundinacea Linn*, which belongs to the *Marantaceae* family, is a perennial plant originating from northern South America. It has been cultivated widely in other tropical areas for its edible tubers, which are the source of arrowroot starch. The arrowroot starches have potential medicinal properties, such as the treatment of gastric ulcers and the protection of the gastrointestinal tract. Damarwati et al. (2020) indicated that arrowroot tuber starch could improve rat stomachs after the ulcer induction [1]. Meanwhile, Gutierrez et al. (2018) suggested that arrowroot starches had a low in vitro digestibility rate, which was beneficial for people suffering from obesity and diabetes [2]. Therefore, arrowroot starches can be applied as raw materials for healthy food products. However, arrowroot starches have been mainly used in starch-based films or baking products so far. Research on value-added utilization of arrowroot starches to create innovative food products is urgently needed.

The 3D printing of foods has potential in precision nutrition and customization of shape or sensory attributes, which can provide enormous opportunities for food standardization and personalization [3]. Food 3D printing can create designated steric morphological constructions of raw food materials via digitally controlled layer-by-layer processes. Techniques of 3D printing usually include extrusion, inkjet printing, and powder binding deposition. Among them, extrusion-based 3D printing is the most widely used one in food processing. After raw materials are extruded by a moving syringe nozzle, three-dimensional food products are formed. Furthermore, 3D printing technology has previously been used in potato, rice and corn starches [4,5]. However, the use of this technology in personalized products of arrowroot starches is still undeveloped. In addition, to meet future business needs, starch gels that are resistant to temperature change still need to be explored.

The 3D printing properties of starch gels are closely related to the physicochemical properties of starches [6]. The physical properties of starches, such as molecular structure, chemical composition, molecular weight, amylose content, gelatinization temperature, crystallinity, viscoelasticity and adhesivity of starch-based materials, and 3D printing conditions can all affect the 3D printing properties of starch gels [4,7]. The chemical composition of starches, such as the contents of proteins, lipids, fibers and polyphenols in natural starches, may influence the gel strength of starch gels, leading to a different 3D printing effect [8]. Zeng et al. (2021) reported that the printability of rice starch gel was improved by catechin and procyanidin [9]. Feng et al. (2018) showed that the addition of 1% pea protein in potato starch enhanced the printing quality through the modulation of interactions between potato starch and pea protein [10,11,12]. However, based on the different sources and extraction methods, there are many differences in the chemical composition (starch, protein, ash, crude fat, crude fiber, etc.), molecular weight, and amylose/amylopectin ratio of arrowroot starches. For instance, arrowroot starches from Kerala contained 12% protein, while those from Yogyakarta contained 23.25% crude fibers [13]. Furthermore, arrowroot starches from various plants were also characterized as A-type, B-type, or C-type crystalline structures, with relative crystallinity ranging from 10% to 30% [13]. During the storage process, the transformation of the crystalline structure or amorphization may occur, resulting in the changes in the physical properties (e.g., flowability, compressibility, and solubility) of arrowroot starches [14]. The relative physicochemical properties of arrowroot starches should be fully characterized before the 3D printing technology was applied. Rheological properties of starch gels, such as thixotropism, viscoelasticity, solidification rate and temperature response during extrusion, are the most important parameters associated with the accuracy and shelf life of 3D printed products [15,16]. However, the above-mentioned rheological properties of arrowroot starch gels have not been fully investigated before [1].

In this paper, arrowroot starch for extrusion-based additive manufacturing applications was firstly investigated. The physical properties of arrowroot starch (e.g., chemical structure, molecular weight, amylose/amylopectin ratio and relative crystallinity) that influence 3D printed features were measured. The overlap concentration, which characterized the transition of arrowroot starch solution from dilute to semi-dilute solution, was determined for the first time. Arrowroot starch gels with different starch concentrations were prepared and evaluated for their rheological properties, including temperature dependence, yield stress, flow stress, frequency dependence and thixotropic properties. The relationship among rheological properties, extrudability and buildability of arrowroot starch gels at different concentrations was analyzed via optimization of the 3D printing parameters. The results provide an optimum condition for the use of arrowroot starches in 3D printing of functional foods.

## 2. Materials and Methods

### 2.1. Materials

Arrowroot starch (AS) was extracted with water (Fengkai, Zhaoqing, Guangdong, China). Chemical composition of AS included 74.3% starch, 15.1% water, 0.1% fiber, 0.3% protein, 0.1% fat, 0.2% ash and 650 mg/kg total flavonoids. The ratio of amylose to amylopectin was 4:11. Milli-Q distilled water (18.3 MΩ) was used in all experiments.

### 2.2. Gel Permeation Chromatography (GPC) Measurements

The molecular weight of arrowroot starch was determined by gel permeation chromatography U3000 (Thermo Fisher Scientific, Waltham, MA, USA), combined with a multi-angle laser light scattering DAWN HELEOS Ⅱ (Wyatt Technology, Santa Barbara, CA, USA), using a differential reflective index detector Optilab T-rEX (Wyatt Technology, Santa Barbara, CA, USA). The chromatographic data were processed by the software ASTRA6.1. The number-average molecular weight (M_n_) and weight-average molecular weight (M_w_) were 1.92 × 10^7^ g/mol and 3.24 × 10^7^ g/mol, respectively. The polydispersity (M_w_/M_n_) was 1.69.

### 2.3. X-ray Diffractometry (XRD) Measurement

The crystal structure of arrowroot starch, which was air-dried at 55 °C for 3 h, was determined by an X-ray diffractometer (Rigaku SmartLab SE, Tokyo, Japan) under the conditions of 40 mA, 40 kV and Cu-K_α_ radiation (λ = 0.154 nm). The scattering angle (2θ) ranged from 5° to 70°, and the scanning speed was 0.02°/min. The degree of crystallinity was calculated using the following equation [17].

Degree of crystallinity (%) = *I_c_*/(*I_c_* + *I_a_*) × 100% (1)

*I_c_* = the integrated area of the crystalline phase;
*I_a_* = the integrated area of the amorphous phase.

### 2.4. Scanning Electron Microscopy (SEM) Measurement

The microstructure of AS granules was studied using a Zeiss Merlin Gemini scanning electron microscope (Ottobrunn, Germany) under 20 kV acceleration voltage. AS granules were fixed on aluminum strips and coated with a thin gold film, and a representative scanning electron microscope of AS was obtained at a magnification of 2000 times.

### 2.5. ^13^C NMR and ^13^C CP/MAS NMR Measurements

^13^C NMR spectra of AS were recorded on a Bruker Advance Ⅲ 500 MHz (^13^C: 126 MHz) NMR spectrometer. AS powder was dissolved in deuterated dimethyl sulfoxide (DMSO)- D_6_ for 1 h, heated in a water bath at 45 °C and 85 °C respectively, and finally placed at room temperature for 1 h. TMS (δ = 0.00) was used as the internal standard for calibrating the chemical shift.

^13^C MAS (magic angle spinning) NMR spectra were recorded on a Bruker Advance Ⅲ 400 MHz (^13^C: 100.62 MHz) spectrometer. The spectrum adopted the cross-polarization (CP) mode, and the self-rotation rate was 10 kHz at a magic angle of 54.7°. The CP contact time was 1.2 ms, the recycle delay was 2 s, and the acquisition time was 0.0254 s. At room temperature, each spectrum accumulated at least 3000 scans.

### 2.6. Differential Scanning Calorimetry (DSC) Measurement

The thermal property of AS was studied by using a DSC 214 Polyma differential scanning calorimeter (NETZSCH-Gerätebau GmbH, Selb, Germany). The AS granules were mixed with distilled water and fixed in an aluminum pot sealed and balanced at room temperature. The analysis was carried out in the temperature range from 30 to 100 °C, with a scanning speed of 10 °C/min.

### 2.7. Preparation of AS Solutions and Gels

To determine the overlap concentration and temperature characteristic scanning of the rheological items, the AS solutions of 0.1–5% (*w/v*) were prepared by stirring and heating in water bath at 45 °C for 10 min. For rheological testing and 3D printing, the aqueous amaranth solution of 10 mg/mL and the aqueous solid green solution of 10 mg/mL were mixed at the ratio of 20:1, then 0.3 mL of the dye mixture was added to the AS gels with concentrations of 10%, 15%, 20%, 25% and 30% (*w/v*), respectively, followed by stirring and heating in a water bath at 85 °C, and finally cooled to room temperature to form gels.

### 2.8. Rheological Properties of AS Solutions and Gels

The rheological properties of AS solutions and gels were performed using an AntonPaar MCR702 rheometer (Graz, Austria). The static rheological tests were carried out using cone plates with diameters of 50 mm, a gap of 0.104 mm, α = 4°, and shear rate of 0.1–1000 s^−1^. The dynamic rheological tests were carried out using parallel plates with diameters of 25 mm. The temperature of AS dispersions or gels was fixed at 25 ± 1 °C. The edges were coated with silicone oil to prevent moisture evaporation.

The linear viscoelastic region was obtained when the strain was in the range of 0.1–100% and the angular frequency was 10 rad/s. Frequency scanning tests were carried out in the viscoelastic range, with the angular frequency ranging from 1 to 100 rad/s.

The gelatinization characteristics were obtained by temperature scanning test under the temperature range from 25 to 100 °C. The temperature stability was obtained by the variable temperature scanning test. The temperature of the three-stage test was set as 25–100 °C/100–25 °C/25–100 °C. The three-interval thixotropy tests (3ITT) were conducted under three-stage variable strains of 1%-100%-1% and at a fixed frequency of 10 rad/s.

### 2.9. The 3D Printing of AS Gels

An extrusion-based 3D printer with a size of 420 × 390 × 482 mm (FOODBOT-S1, Shiyin Technology Co., Ltd, Hangzhou, China) was used to study the effects of different nozzle diameters, printing/extrusion speeds, temperatures and AS concentrations on the printing accuracy of AS gels. The printed model was designed by a 3D modeling software (Pro/ENGINEER, version 5.0, Boston, MA, USA), and the 3D models were analyzed and exported by the Repetier Host software. The sizes of the printed samples were measured with a ruler, and the results of three repeated measurements were analyzed by Statistical Product and Service Solutions software (version 20.0, Chicago, IL, USA).

### 2.10. Statistical Analysis

The data were expressed as mean ± standard deviation. The Duncan test and one-way analysis of variance (ANOVA) were used to analyze the significant difference between the data using SPSS20.0 software. If *p* < 0.05, the difference was considered as statistically significant.

## 3. Results and Discussion

### 3.1. ^13^C NMR Analyses

**Figure 1:**
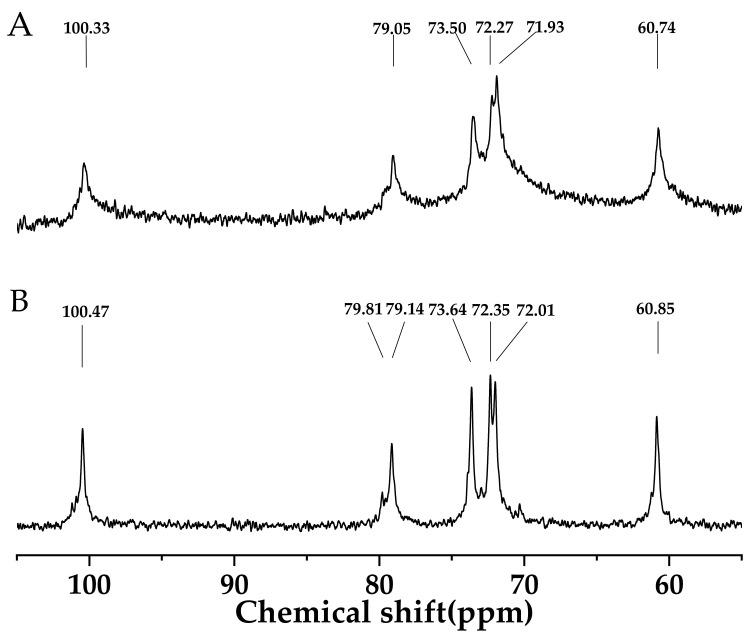
(**A**) ^13^C NMR spectrogram of arrowroot starch treated at 45 °C and (**B**) ^13^C NMR spectrogram of arrowroot starch treated at 85 °C. Here, deuterated dimethyl sulfoxide (DMSO- D_6_) was used as the solvent.

^13^C NMR spectroscopy of AS treated at 45 °C was shown in Figure 1. The signals located at 100 ppm, 71–80 ppm and 60 ppm corresponded to C-O anomeric, CH-O and CH_2_-O, respectively. The chemical shifts of AS at peak values of 100.33, 79.05, 73.50, 72.35, 71.93 and 60.74 ppm represented C-1, C-4, C-3, C-2, C-5 and C-6, respectively [18]. C-4 was assigned to non-reducing terminal units, and C-6 represented the α-1-6 linkages linking (1-4)-α-D-glucans. All sugar residues in the AS were in pyranose form and attributed to the absence of signals between 80 and 88 ppm. This structural profile of AS in ^13^C NMR spectroscopy was similar to those of waxy-maize and wheat starch, but different from inulin-type polysaccharides [19,20]. Figure 1B showed the ^13^C NMR spectrum of AS treated at 85 °C. Characteristic peak values of AS slightly shifted to the higher position, probably due to the intra- or inter- molecular interaction. Intra- or inter-molecular hydrogen bonding might appear between the CH_2_-O and the adjacent hemiacetal oxygen atom of the D-glucopyranosyl residues, or between the CH-O and the adjacent CH_2_-O of the D-glucopyranosyl residues on different molecules of amylose, which promoted the formation of the spatial network structure [21].

### 3.2. Scanning Electron Microscopy (SEM) Analysis

**Figure 2:**
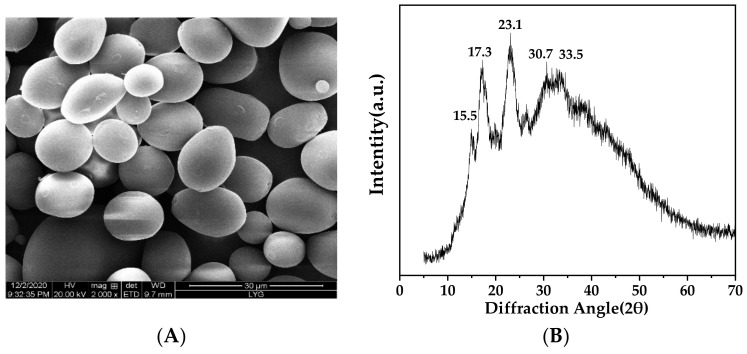

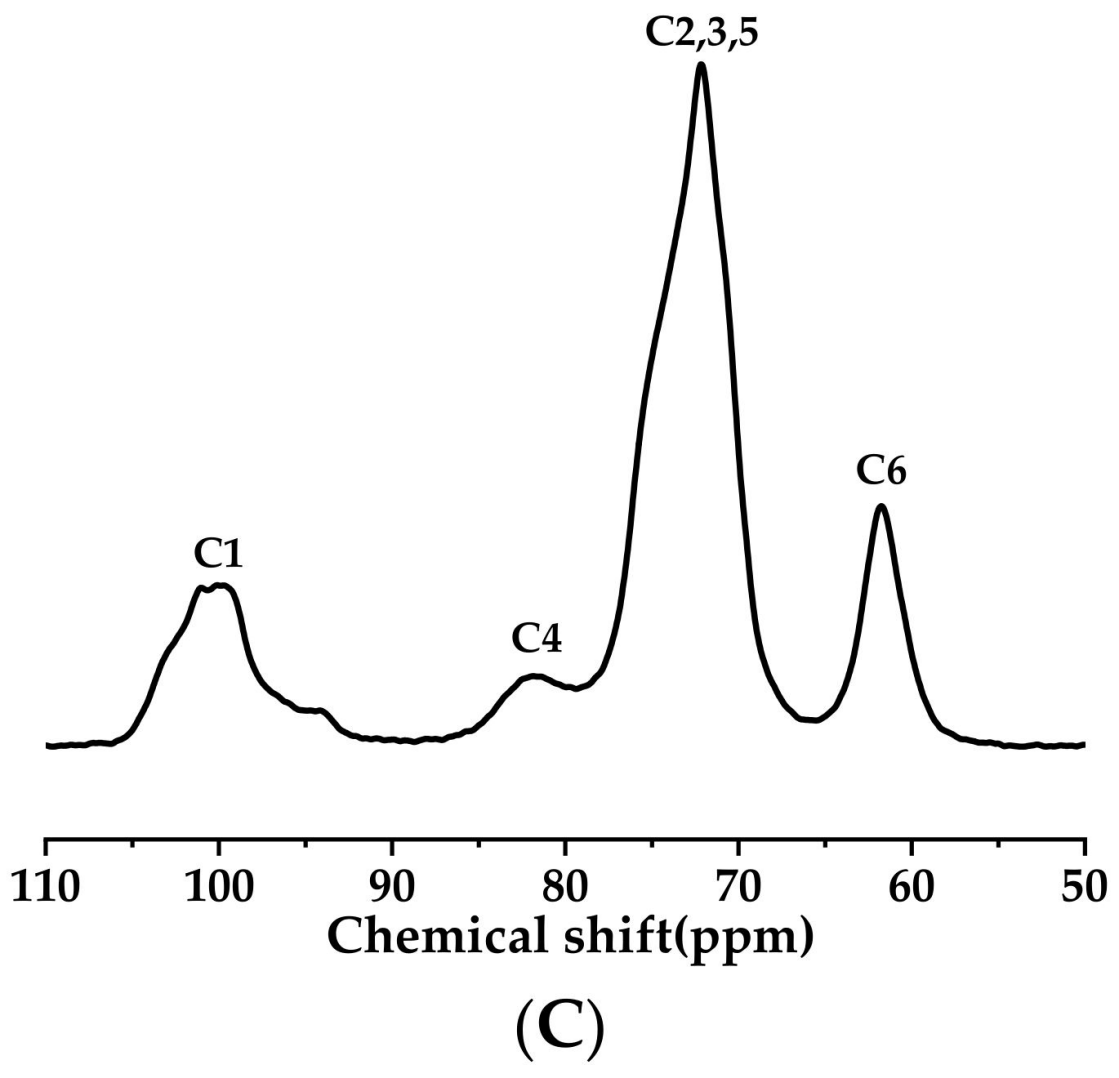
(**A**) SEM micrograph of arrowroot starch (magnification was 2000); (**B**) X-ray diffraction pattern of arrowroot starch; and (**C**) ^13^C Solid-State NMR of arrowroot starch powder.

Figure 2A showed the SEM image of AS granules. The morphology of AS granules was a compact spherical or elongated shape, which was probably related to the high amylopectin content of AS (amylose/amylopectin ratio of 4:11). Similar morphology was observed in potato starch granules (the moisture content of 18.3%, the amylose content of 23.7%, *w/w*) [22,23]. The sizes of AS granules ranged from 6 to 18 μm with an average size around 10.5 ± 2.5 μm, which was larger than that of arrowroot starch from India, but much smaller than that of native potato starch [24].

### 3.3. X-ray Diffraction Analysis

Arrowroot tuber starch derived from different regions exhibited a distinctive semi-crystalline structure [25,26,27]. The XRD pattern of AS is shown in Figure 2B. The major diffractions at 2θ = 15.5°, 17.3° and 23.1° with an additional diffraction of 2θ = 30.7° and 33.5° was assigned to the A-type crystalline structure of AS [14]. The relative crystallinity of AS was calculated as 26.5%. The A-type crystalline pattern of AS was ascribed to the short branch chains of amylopectin [18], which contributed to the semi-crystalline structure in AS granules. A more rigid structure attributed to the short branch chains of amylopectin might prevent amylopectin double helices from aligning [28]. Zeng et al. (2021) suggested that the compact and inhomogeneous crystalline network structure could help improve the mechanical strength (G′, τ_y_) of starch gels [9,29]. Accordingly, the crystallinity value is important structural information affecting the fluidity, accuracy and adhesivity of 3D printing of AS.

### 3.4. ^13^C CP/MAS NMR Analysis

Solid state ^13^ C CP/MAS NMR spectrum of AS granules was shown in Figure 2C. The resonance peaks were divided into C-1 region of 99 to 102 ppm, C-2, C-3 and C-5 regions of 68 to 77 ppm, C-4 resonance peak of 78.6 ppm and C-6 region of 60 to 65 ppm [30]. The fitted triplet state of the C-1 region was assigned to the helical structure of the three arranged glucose residues, suggesting the A-type crystalline structure of AS, which was consistent with the XRD analysis [30]. The single helices peaks and double helices appeared at 102–103 ppm and 90–102 ppm, respectively. Therefore, the C1 region of 99–102 ppm referred to double-helical components in AS granules [31]. The ordered structure and nonordered structure were 37.7% and 41.4%, respectively [32,33]. Liu et al. (2020) suggested that the ordered structure of potato starch samples influenced the gelatinization temperature and effect of 3D printing [34]. The difference in the ordered structure between the AS and potato starch samples might contribute to the distinct results of 3D printing.

### 3.5. Rheological Properties of AS Solutions and Gels

#### 3.5.1. Overlap Concentration of AS Solution

**Figure 3:**
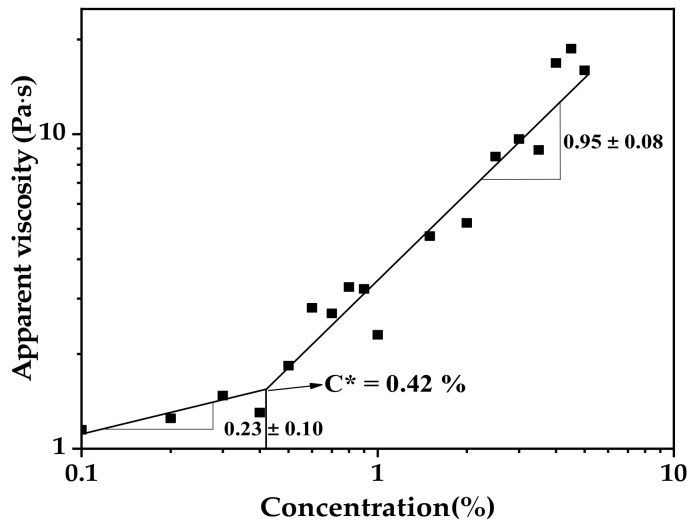
Plot of apparent viscosity versus concentration for arrowroot starch aqueous solution. The overlap concentration C* was the crossover point of the two fitted lines.

The overlap concentration (C*) is the boundary between the dilute and semi-dilute regimes of a polymer solution. C* occurs when the polymer chains start to overlap with each other [35]. Figure 3 showed the fitting curve of the apparent viscosity (η_sp_) versus the concentration of AS aqueous solution ©. The C* value of 0.42% (*w/v*) in AS aqueous solution was determined from the crossover point of the two fitted lines. The C* value of AS dispersion was similar to that of the potato starch (0.43%, *w/v*), but lower than the C* value of the sweet potato starch (0.54%, *w/v*) [36]. Guo et al. (2016) suggested that C* is inversely proportional to the molecular weight and amylopectin content [36]. When the concentration of AS solution was higher than C*, AS molecules overlapped and penetrated, causing the solution viscosity to increase dramatically.

#### 3.5.2. Thermal and Rheological Properties of AS Gels

**Figure 4:**
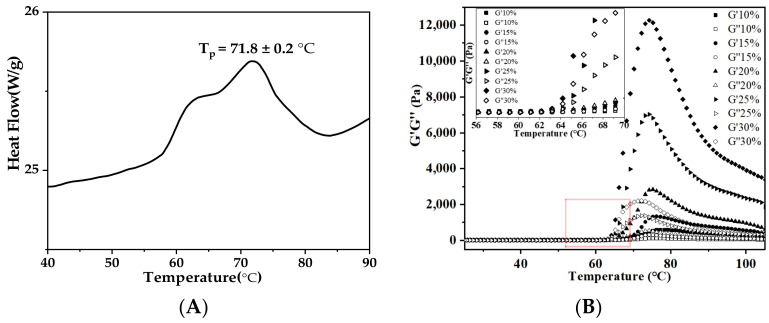

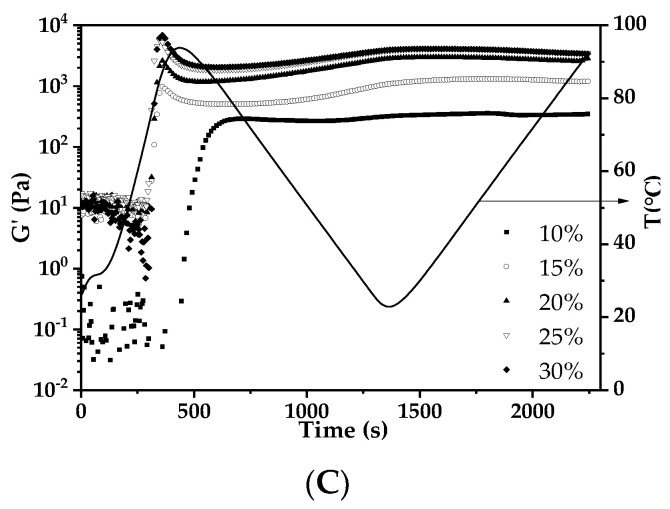
(**A**) Differential scanning calorimetry thermogram of arrowroot starch; (**B**) plots of storage modulus (G′) and loss modulus (G′′) versus temperature for arrowroot starch samples with concentrations ranging from 10% to 30%; and (**C**) effect of variable temperature scanning (heating → cooling → heating cycle) on storage moduli of arrowroot starch samples with concentrations ranging from 10% to 30%.

Thermal and rheological analyses are critical to the understanding of the structure-function relationship of AS in aqueous solution during gelatinization. The differential scanning calorimetry (DSC) results indicated that the onset (T_o_), peak (T_p_), conclusion (T_c_) temperatures and enthalpy change (∆H) were 57.9 ± 0.1 °C, 71.8 ± 0.2 °C, 78.9 ± 0.8 °C and 16.3 ± 1.4 (J/g), respectively, as shown in Figure 4A. Dynamic rheological properties of AS gels during the temperature variation from 25 °C to 100 °C were performed using a MCR702 rheometer. AS with different concentrations exhibited similar profiles of viscoelasticity (Figure 4B). Storage modulus (G′) increased sharply to a maximum between T_o_ and T_c_, and decreased upon further heating [37]. When the temperature was above T_o_, the crystal structure of AS was destroyed, and the network structure began to form; the values of tanδ were lower than 0.2 [38]. After the temperature reached T_p_, the formation of AS gels led to the highest G′ value. The strength of hydrogen bonding between water and AS molecules strongly depended on starch source [39]. The T_G′max_ values of AS of different concentrations were significantly different from those of potato, corn and rice starches [38,40]. Within this temperature range, G′ increased with the increase in AS concentration. Because the concentration of AS was far beyond C*, the network structure formed, owing to the entanglement between amylose and amylopectin [6]. As the temperature continuously increased, the drop in G′ value could be attributed to the destruction of the intermolecular interactions and the breakdown of chain entanglements.

Figure 4C exhibits the impacts of temperature ramping on the storage modulus (G′) of AS gels before and after gelatinization. Before gelatinization, when the temperature was first ramped up from 25 °C to 100 °C, the G′ values increased quickly. It took less time for the G′ to reach the maximum at the higher AS concentration. After gelatinization, the G′ values of all the AS gels studied were negligibly affected by temperature ramping down (second stage, 100–25 °C) or ramping up (third stage, 25–100 °C). During the whole temperature variation stages, the G′ value of 10% AS gel was lower than 500 Pa, while the AS gels with the concentrations of 15% to 30% were all higher than 500 Pa. The AS gels of higher concentrations consistently showed a larger G′ plateau value [41]. In general, the exclusion of moisture in starch gels and re-crystallization may increase the rigidity of starch gel with the decrease in temperature. These properties may affect the expanding volume and the stability of 3D printed products.

**Figure 5:**
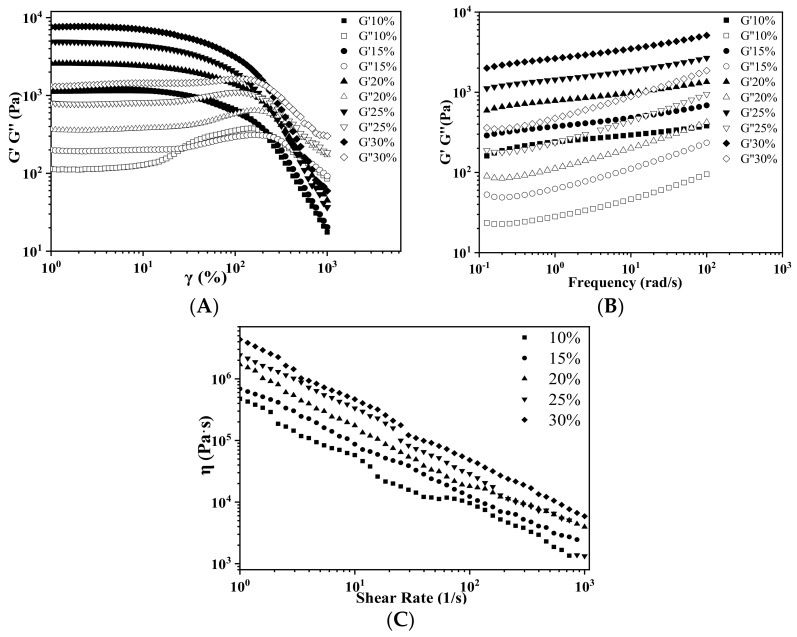
(**A**) Plots of storage modulus (G′) and loss modulus (G′′) versus strain for arrowroot starches of different concentrations; (**B**) plots of storage modulus (G′) and loss modulus (G′′) versus angular frequency for arrowroot starches of different concentrations; and (**C**) plots of apparent viscosity versus shear rate for arrowroot starches of different concentrations.

Figure 5A shows the stress sweep results of AS gels of different AS concentrations. The G′ values were related to the hardness of gels, the yield stress (τ_y_) represented the mechanical strength of materials for supporting the subsequently deposited layers and maintaining the printed shapes, while the flow stress (τ_f_) reflected the extrudability of AS gel during 3D printing. With the increase in AS concentrations, the G′, τ_y_ and τ_f_ values all increased. τ_y_ and τ_f_ increased from 219.3 Pa to 1422.0 Pa and from 1134.7 Pa to 6909.9 Pa, respectively. The higher the τ_y_, the better resistance to deformation and the better the printing accuracy; while the higher the τ_f_, the greater force was needed during 3D printing. This tendency was consistent with that of rice, potato or corn starches [34,38]. However, different starch gels had different G′, τ_y_ and τ_f_ values, which were determined by the type and chemical structure of starches, as well as the starch concentration and pasting condition [42]. With the increase in AS concentration, the gel network structure became denser and thicker, which was influenced by the microstructure of different starches, leading to the different G′, τ_y_ and τ_f_ values [9,43].

Figure 5B exhibited frequency dependence of the dynamic moduli of AS gels with different concentrations. Over the whole frequency and AS concentration ranges studied, the G′ values were consistently higher than the G′′ values, suggesting the gel-like behaviors of these AS samples. These results may further indicate the stability of the AS gels under a rapid movement at a short time or under a slow movement or static state in a long time.

Shear-thinning of materials is a key factor of 3D printing. Figure 5C showed the apparent viscosities of AS gels at different AS concentrations, which exhibited pseudoplastic fluids with shear-thinning behavior. This result suggested that AS gel could be easily extruded from the printer nozzle under an appropriate shear force.

**Figure 6:**
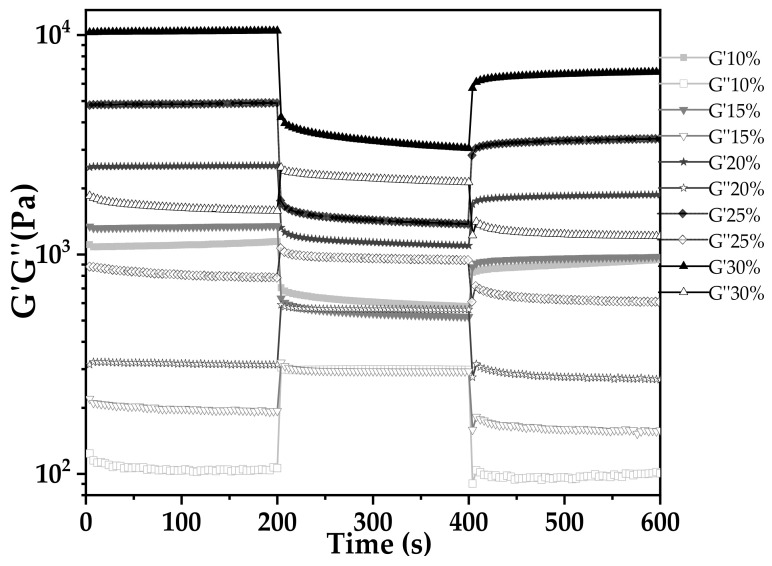
Three-interval thixotropy test of arrowroot starch samples with concentrations ranging from 10% to 30% (*w/v*).

The capacity of rapid structure recovery following extrusion is another critical factor for 3D printing. Figure 6 shows the thixotropy behaviors of AS gels at different concentrations. There were three stages of this test. The first stage (low shear stress) was a simulation of AS gel loading in the feed cylinder of the printer. The second stage (high shear stress) simulated the extrusion and printing process. The third stage (low shear stress) was a simulation of gravity action on AS gel after printing [38]. The G′ values were higher than G′′ at either high or low shear stress, suggesting that the viscoelastic properties were maintained. As shown in Table 1, the recovery degree of the material was evaluated by the ratio of the average G′ of the third stage to the first stage (G′_3_/G′_1_). In combination with the result of 3D printing (Section 3.6.4), it was found that although the recovery degree of 10% AS is 80%, its G′ was always at a relatively low level. Due to its weak mechanical strength, the printed AS gels had poor self-supporting performance and easily collapsed after 3D printing. For 30% AS gel, its mechanical strength was too high (G′ ≥ 10,000 Pa) for the materials to be extruded. The relatively low recovery degree (60%) for 30% AS gel also led to a poor printing effect. It should be noted that the optimum printing precision of AS gels occurred at 20% AS concentration, which has a recovery degree of 71% and a storage modulus in the range of 2000–5000 Pa.

### 3.6. The 3D Printing of AS Gels

#### 3.6.1. Effect of Nozzle Size

The nozzle size is an important factor affecting the ductility of AS gels. Four nozzle diameters of 0.41, 0.60, 0.84 and 1.20 mm were chosen for 3D printing (Table 2). Due to the good ductility and stress restorability of AS gels (Figure 6), AS gels could be printed using various nozzle sizes. However, based on the accuracy of the printed products, the 0.60 mm nozzle diameter was found to provide the optimal results. In contrast, products printed using a nozzle diameter of 0.41 mm had an irregular surface pattern, which may be associated with the insufficient filling caused by the incomplete extrusion. Products printed using the nozzle diameter of 1.20 mm had unclear textures due to the extrusion overflow [44,45].

#### 3.6.2. Effect of Printing/Extrusion Speed

In order to establish the dynamic work range of the 3D printer for AS gels, the effect of the printing/extrusion speed (mm/s) with 80/80, 80/100, 100/80, 100/100, 100/120, 120/100 and 120/120 mm/s on the printing process was analyzed (Table 3). When the printing speed was higher than the extrusion speed (100/80 mm/s), the supply speed of the AS gel was insufficient, leading to the breaking of the printing layers. When the printing speed exceeded 100 mm/s and the extrusion speed was 20 mm/s higher than the printing speed, the overflow of AS gels caused higher printing layers, which significantly influenced the printing accuracy [46]. Accordingly, the printing speed of 100 mm/s and the extrusion speed of 100 mm/s were the optimal printing/extrusion speeds for the 3D printing of AS gels, which could be demonstrated by the high G′ value (Figure 5A) and G′ > G′′ in the high shear stage (Figure 6). For the starch gels of high G′ values, a balance of the printing and extrusion rate is highly desired. However, the filling of the 80/80 mm/s is insufficient, and the printing is incomplete. The printing accuracy of 120/120 mm/s is high, but the printing size is quite different from the original one.

#### 3.6.3. Effect of Temperature

Table 4 shows the effect of printing temperatures in the range of 25 to 85 °C on the 3D printing of AS gels. Within this range, temperature had little effect on the printing accuracy. This result is consistent with the finding that the G′ values of AS samples after gelatinization remain relatively constant during the third stage (25–100 °C) of the temperature variation test, as reported in Figure 4C. This phenomenon might be attributed to the high molecular weight and high amylopectin content of AS. It took a long time for the retrogradation and dehydration of the AS gel, due to the presence of amylopectin with a large number of short side chains [21]. In contrast, when the printing temperature is equal to or above 75 °C, the less effective interactions between starch chains of the potato starch gel were attributed to weakened mechanical strength and poor printability [34]. This phenomenon might be associated with the phosphate ester groups and ordered structure of potato starch.

#### 3.6.4. Effect of AS Concentration

Table 5 shows the effect of AS concentrations on 3D printing. When the AS concentration was 10%, the printed product collapsed during printing, which could be due to the low apparent viscosity in the rheology test (Figure 5C). When the AS concentration reached 30%, the G′ value was too high, resulting in poor material flow under extrusion. In addition, a higher viscosity led to the reduction in printing accuracy and the brittleness of the printed products [47]. The good structural performance and stability of the printed products occurred at AS concentrations ranging from 15% to 20%. The product with the highest accuracy and stability was found at 20% AS gel. These results indicated that the concentration of AS directly affected the formation and strength of the gel network structure, further affecting the gel printing performance and stability [48]. The optimal printing concentration of AS samples depended on both rheological properties and microstructure of the starch gels [38].

## 4. Conclusions

In this study, the physicochemical, morphology, and structural properties of arrowroot starch (AS) were investigated. The morphology of AS granules was a compact spherical or elongated shape. Both XRD patterns and ^13^C CP/MAS NMR spectra revealed that AS had an A-type crystal structure. The dynamic rheological measurements of AS showed good structural recovery and thermal stability, and the mechanical strength increased with the increase in AS concentration. Therefore, the printing quality of AS samples after gelatinization was stable over a wide range of printing temperatures, ranging from 25 to 85 °C. The optimum printing effect occurred when the AS concentration (20%) was 50 folds higher than C* (0.42 %). However, when the AS concentration was too high (i.e., 70–72 times of C*), the AS gel was brittle and had improper material flow during the extrusion process. Since the AS gels had good ductility and restorability under stress, AS gels could be printed successfully using different nozzle diameters. Among them, the 3D printed AS gels showed the best accuracy and appearance when the nozzle diameter was set at 0.60 mm. When the printing speed was higher than the extrusion speed or the extrusion speed was greater than printing speed, the breaking of printing layers or overflow of AS gels may occur. Overall, the printing speed of 100 mm/s in combination with the extrusion speed of 100 mm/s was the optimum printing/extrusion speed for the 3D printing of AS gels. Although the molecular weight and temperature gelatinization of AS, as well as G′ of AS gel, were much higher than those of potato starch gels, the optimal printing concentration of these two kinds of starches were similar. Since natural AS gel without any additive is thermally resistant and suitable for 3D printing, AS gels can be used as the delivery vehicle for personalized nutrition control. Further research on 3D printed products of AS gels being used as a carrier of functional compounds is currently being carried out in our laboratory. The results obtained from this study can not only help us understand the structure and function relationships of arrowroot starches, but also provide a valuable guidance for them to be used in the design of novel personalized functional food products.

## Figures and Tables

**Table 1:** Parameters during the three-interval thixotropy tests (3ITT) for AS concentrations ranging from 10 to 30% (*w/v*).

Variety	Concentration (%)	G′_1_ (Pa)	G′_3_ (Pa)	G′_3_/G′_1_ (%)
AS	10	1107.6	887.6	80%
	15	1336.2	927.3	69%
	20	2527.4	1783	71%
	25	4844.5	3090.4	64%
	30	10,371.9	6289.4	60%

**Table 2:** Effect of nozzle diameter on 3D printing of AS gels.

Nozzle Diameter (mm)	Length (mm)	Width (mm)	Height (mm)	Print Shape
Model	40	40	5	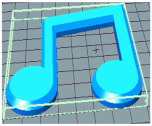
0.41	38.7 ± 1.5 ^b^	39.0 ± 1.0 ^a^	4.6 ± 0.1 ^d^	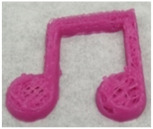
0.60	41.0 ± 1.0 ^a^	40.5 ± 0.5 ^a^	5.1 ± 0.1 ^c^	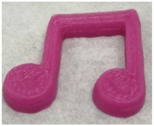
0.84	41.0 ± 1.0 ^a^	40.2 ± 0.5 ^a^	5.9 ± 0.1 ^a^	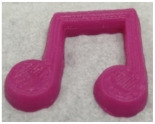
1.20	40.5 ± 0.5 ^b^	38.7 ± 1.5 ^a^	5.7 ± 0.1 ^b^	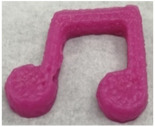

Note: the values of different letters in the same column are significantly different at the *p* < 0.05 level. The printing temperature is 25 °C, the printing/extrusion speed is 100/100 mm/s and the print concentration of AS is 20% (*w/v*).

**Table 3:** Effect of printing/extrusion speed on 3D printing of AS gels.

Printing/Extrusion Speed (mm/s)	Length (mm)	Width (mm)	Height (mm)	Print Shape
Model	35	35	5	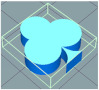
80/80	32.3 ± 0.8 ^c^	29.6 ± 1.1 ^c^	4.6 ± 0.4 ^ab^	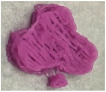
80/100	34.8 ± 0.2 ^a^	34.8 ± 0.2 ^b^	4.4 ± 0.1 ^b^	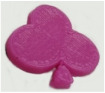
100/100	34.9 ± 0.1 ^a^	34.9 ± 0.1 ^a^	4.9 ± 0.7 ^a^	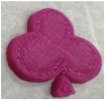
120/100	34.4 ± 0.4 ^a^	35.0 ± 0.2 ^a^	4.8 ± 0.2 ^a^	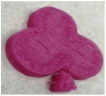
100/80	33.5 ± 0.5 ^b^	34.2 ± 0.2 ^a^	3.7 ± 0.1 ^c^	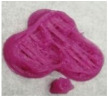
100/100	34.9 ± 0.1 ^a^	34.9 ± 0.1 ^a^	4.9 ± 0.7 ^a^	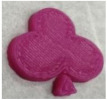
100/120	35.0 ± 0.2 ^a^	34.9 ± 0.7 ^a^	5.0 ± 0.4 ^a^	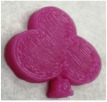
120/120	34.3 ± 0.4 ^a^	33.3 ± 0.3 ^b^	4.9 ± 0.1 ^ab^	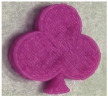

Note: the values of different letters in the same column are significantly different at the *p* < 0.05 level. The nozzle diameter is 0.60 mm, the printing temperature is 25 °C, and the print concentration of AS is 20% (*w/v*).

**Table 4:** Effect of temperature on 3D printing of AS gels.

Printing Temperature (°C)	Length (mm)	Width (mm)	Height (mm)	Print Shape
Model	28	28	5	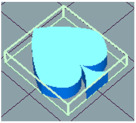
25	27.7 ± 0.3 ^a^	27.9 ± 0.8 ^a^	5.0 ± 0.1 ^a^	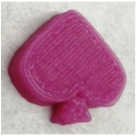
37	28.0 ± 0.2 ^a^	28.0 ± 0.3 ^a^	5.1 ± 0.6 ^a^	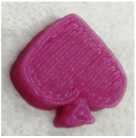
45	28.0 ± 0.8 ^a^	27.6 ± 0.1 ^b^	5.1 ± 0.1 ^a^	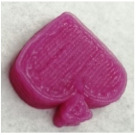
60	27.8 ± 0.2 ^a^	27.9 ± 0.7 ^a^	5.1 ± 0.6 ^a^	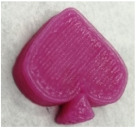
75	28.0 ± 0.1 ^a^	28.0 ± 0.1 ^a^	5.1 ± 0.5 ^a^	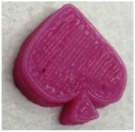
85	27.7 ± 0.2 ^c^	27.1 ± 0.1 ^c^	5.3 ± 0.3 ^a^	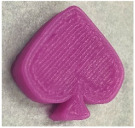

Note: the values of different letters in the same column are significantly different at the *p* < 0.05 level. The nozzle diameter is 0.60 mm, the printing/extrusion speed is 100/100 mm/s, and the print concentration of AS is 20% (*w/v*).

**Table 5:** Effect of AS concentration on 3D printing of AS gels.

AS Concentration (%)	Length (mm)	Width (mm)	Height (mm)	Print Shape
Model	30	30	20	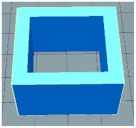
10	26.9 ± 0.6 ^b^	23.3 ± 1.0 ^b^	14.8 ± 0.2 ^b^	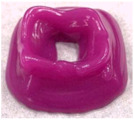
15	30.3 ± 1.0 ^a^	28.6 ± 1.3 ^a^	20.2 ± 0.9 ^a^	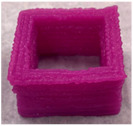
20	30.0 ± 1.0 ^a^	28.7 ± 0.6 ^a^	20.5 ± 0.4 ^a^	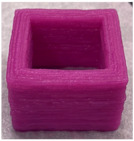
25	29.4 ± 0.9 ^a^	29.0 ± 1.0 ^a^	20.7 ± 0.2 ^a^	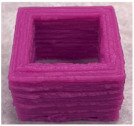
30	28.8 ± 0.6 ^a^	28.0 ± 1.0 ^a^	20.3 ± 0.3 ^a^	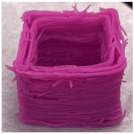

Note: the values of different letters in the same column are significantly different at the *p* < 0.05 level. The nozzle diameter is 0.60 mm, the printing temperature is 25 °C, and the printing/extrusion speed is 100/100 mm/s.

## Data Availability

The data presented in this study are available on request from the corresponding author.
